# Perturbed oral motor control due to anesthesia during intraoral manipulation of food

**DOI:** 10.1038/srep46691

**Published:** 2017-04-20

**Authors:** Joannis Grigoriadis, Abhishek Kumar, Peter Svensson, Krister G. Svensson, Mats Trulsson

**Affiliations:** 1 Section of Oral Rehabilitation, Department of Dental Medicine, Karolinska Institutet, Huddinge, Sweden; 2 Scandinavian Center for Orofacial Neurosciences (SCON), Huddinge, Sweden; 3 Section of Orofacial Pain and Jaw Function, Department of Dentistry, Aarhus University, Aarhus, Denmark

## Abstract

Sensory information from periodontal mechanoreceptors (PMRs) surrounding the roots of natural teeth is important for optimizing the positioning of food and adjustment of force vectors during precision biting. The present experiment was designed to test the hypothesis; that reduction of afferent inputs from the PMRs, by anesthesia, perturbs the oral fine motor control and related jaw movements during intraoral manipulation of morsels of food. Thirty healthy volunteers with a natural dentition were equally divided into experimental and control groups. The participants in both groups were asked to manipulate and split a spherical candy into two equal halves with the front teeth. An intervention was made by anesthetizing the upper and lower incisors of the experimental group while the control group performed the task without intervention. Performance of the split was evaluated and the jaw movement recorded. The experimental group demonstrated a significant decrease in measures of performance following local anesthesia. However, there was no significant changes in the duration or position of the jaw during movements in the experimental and control group. In conclusion, transient deprivation of sensory information from PMRs perturbs oral fine motor control during intraoral manipulation of food, however, no significant alterations in duration or positions of the jaw during movements can be observed.

Masticatory jaw movements during biting and chewing of food morsels, are modulated by afferent sensory inputs from different orofacial tissues^1^,^2^,^3^. These tissues contain several different microstructures or thin nerve endings which function as receptors and are categorized into proprioceptors, nociceptors and exteroceptors^4^,^5^,^6^. Adequate function of the jaw motor system relies on sensory information gathered from the sensory receptors in the orofacial region. One such important and specialized receptor called the periodontal mechanoreceptor (PMR), is imbedded in the periodontal ligament (a dense collagenous tissue) extending along the roots of the teeth. The PMRs provide important information to the central nervous system (CNS) regarding the levels and directions of the force, position of the food and spatial orientation during the initial tooth-food contact^7^,^8^,^9^. The afferent sensory information from the PMRs is projected to the primary somatosensory cortex (SI), where it is integrated and interpreted and then further coordinated in the primary motor cortex (MI) to generate an efferent motor command to different jaw muscles^4^,^10^. It seems likely that the reduction in the sensory information provided from these mechanoreceptors may perturb oral motor control and thus impair mastication^11^,^12^.

We have previously proposed that PMRs play a key role in positioning and splitting morsels of food with considerable accuracy/precision^13^,^14^,^15^,^16^. Manipulation of such morsels by individuals with fixed implant-supported prostheses lacking the periodontal ligament (no PMRs); is perturbed^14^. Therefore, we hypothesized that sensory information from the PMRs surrounding the roots of natural teeth is important for optimizing the positioning of food and adjustment of force vectors during precision biting. It could be anticipated that transient deprivation of sensory inputs in natural dentate participants (due to local anesthesia) would result in perturbed behavior similar to those with fixed implant-supported prostheses. Hence, the aim of the present experiment was to test the hypothesis; that decreased inputs from the PMRs, due to local anesthesia, would perturb the oral fine motor control and the related jaw movements, during intraoral manipulation of food morsels.

## Materials And Methods

### Participants

The study was preapproved by the regional ethical review board in Stockholm, Sweden (Dnr 2012/1562-31/1). Participation was voluntary and all participants provided their written informed consent prior to the start of the experiment. Further, they were also informed of their right to discontinue the experiment whenever they wanted. All experiments were performed in accordance with the relevant guidelines and regulations in accordance with the Declaration of Helsinki.

Thirty young healthy volunteers, who had also participated in a previous study wherein, they performed the same behavioral task (see below) for approximately 30 minutes, were recruited^15^. These participants, who exhibited healthy natural dentition (no known periodontal disease) in both their upper and lower jaws, were divided into an experimental group (five men and ten women, 27 (23–32) years of age (mean (range))) and a control group of equal size (nine men and six women, 25 (21–29) years of age). None of the participants reported any orofacial pain or associated disturbed jaw function. A simple intraoral screening revealed that participants did not have any ongoing or prior evidence of prosthodontic and endodontic treatment during the time of the experiment. In addition, the participants were only included if they did not have any fixed retainers or ongoing orthodontic treatment.

### Recording of jaw movements, electromyographic activity and sounds

The participants were seated in an office chair, in an upright position without any head support, and their jaw movements recorded while performing the behavioral task as described in detail previously^12^,^14^,^17^,^18^. In brief, the movement of the lower jaw in relationship to the upper was assessed in three-dimensions employing a small gold-plated magnet (10 × 5 × 10 mm; Neodymium Iron Boron, N42) attached to the labial surface of the lower central incisors with dental composite. The position of this magnet was tracked with the help of eight sensors (four on each side; accuracy: 0.1 mm; bandwidth: DC – 100 Hz) attached to a light weight wooden frame (220-gram; Umeå University, Physiology Section, IMB, Umeå, Sweden) resting on the bridge of the nose like a pair of spectacles and anchored to the head with adjustable straps.

**Figure 1:**
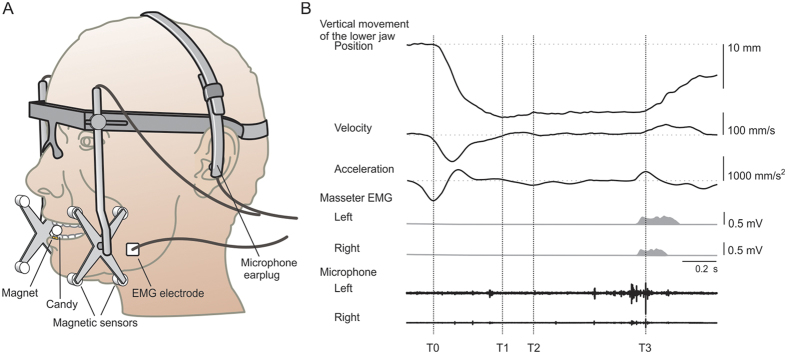
(**A**) The device custom built to monitor movement of the lower jaw relative to the upper jaw during different behavioral tasks. Magnetic sensors (four on each side) located on arms projecting down from the frame track the position of a magnet attached to the labial surface of the lower incisors. EMG activity was recorded bilaterally from the masseter muscles using bipolar surface electrodes. Sounds pertaining to crackling of the candy were recorded bilaterally by microphones secured in an earpiece on a headgear. (**B**) Representative recordings made during the “manipulation and split” task performed by a single participant. From top to bottom the curves depict: *position*; *velocity* and *acceleration* of the vertical movement of the jaw; muscle activity (the r.m.s.-processed EMG) from the left and right masseter muscles; and sound recordings from the left and right ear microphones. The events of interest are the following: The onset of the *jaw opening phase* (T0). *End of the opening phase*, and *start of the contact-establishing phase* (T1). *End of the contact-establishing phase*, and *start of the contact phase* (T2). *End of the contact phase*, and *start of the jaw-closing phase* (T3). The splitting of the candy was detected as rapid closing of the jaw that coincided with both a clear sound and increased EMG activity.

Electromyographic activity (EMG) from the masseter muscles were also recorded bilaterally using shielded pre-amplifiers (bandwidth: 6 Hz to 2.5 kHz) mounted on the skin directly above the surface electrodes (2 mm in diameter and 12 mm apart, custom build at Umeå University, Physiology Section, IMB, Umeå, Sweden). The masseter muscle was palpated by asking the participant to clench their teeth and then the electrodes were placed on the most prominent part of the muscle, perpendicular to the direction of the muscle fibers. The skin over the surface of the masseter was cleaned with alcoholic wipes (99.5% ethanol). The electrodes were coated with electrode gel and secured to the skin with double adhesive tapes. The sound pertaining to the crackling of the food morsel during the behavioral task was recorded by microphones secured in an earpiece on a headgear (custom build at Umeå University, Physiology Section, IMB, Umeå, Sweden) and placed in the external ears (see, Fig. 1A).

### Anesthetic intervention

Intervention with injections of local anesthetic solution around the roots of upper and the lower central/lateral incisors was performed in the experimental group after 30 repetitions of the behavioral task. However, the participants in the control group continued to perform the behavioral task without any intervention (no control injections). The central and lateral incisors were anesthetized in both the upper and lower jaws by local infiltration of approximately 2 × 1.8 ml anesthetic solution (Citanest^®^ Dental Octapressin^®^ (1.8 ml cartridge; Prilocain-hydroclorid (30 mg/ml) and Felypressin (0.54 mg/ml), Dentsply Ltd, Umeå, Sweden) in the buccal sulcus. The participants confirmed the subjective symptoms associated with local anesthesia and objectively it was assessed by lack of response to light touch, pressure to the teeth and gingiva in the region of anesthetized tissues.

### The behavioral manipulation task

The behavioral task here was the same “manipulation and split” task described previously in detail by Svensson *et al*.^14^ and Kumar *et al*.^15^. In brief, the participants were asked to place a spherical sugar-coated chocolate candy (10 mm in diameter, 0.84 g; Fazer Marianne chocolate dragees, Fazer konfektyr AB, Stockholm, Sweden) between the midsection of the palate and the tongue and subsequently move this candy in between the anterior incisors and attempted to split it into two equal halves. It may be noted that no information was given to the participants regarding how quickly the task should be performed. The participants repeated the behavioral task 30 times before and after the intervention (in total 60 repetitions during the experiment).

### Data acquisition and analysis

The performance was evaluated by comparing the weight of the largest piece resulting from the split to half the weight of the candy (0.42 g), with a precision of ±0.01 g (Fino Balance Mini; Fino GmbH, Bad Blocket, Germany)^14^,^15^. The smaller the deviation from the ideal split the better the performance^14^. A split was characterized as ‘*ideal*’ if the deviation from half the weight of the candy was zero percent (i.e., largest piece after split had a weight of 0.42 g). Further, if the deviation from ideal split of the candy was >50% such a split was characterized as ‘*unsuccessful*’ split. Whereas, if the deviation was >75% then the split was characterized as ‘*failed*’ split. Furthermore, those splits with a deviation <5% were characterized as ‘*perfect*’ splits.

Data regarding the jaw movements was recorded using a computer based acquisition and analysis software (WinSc/WinZoom v1.54; Umeå University, Physiology Section, IMB, Umeå, Sweden). The jaw movements where sampled at a frequency of 800 Hz, EMG signals sampled at 3.2 kHz and sound related to crackling of the candy was recorded at 25.6 kHz. Several points of interest during the individual trials were identified by the software and checked manually for errors. These points of interest were the *onset of jaw opening* (i.e., T0) which was defined as the time-point at which the vertical acceleration at the *beginning of jaw opening* was at maximum (i.e., the first peak negative value). The *end of jaw opening phase* (T1) was determined when the vertical velocity exceeded zero for the first time (*beginning of the contact-establishing phase*) and then subsequently exceeded zero thereafter; marked as the *end of contact-establishing phase* (T2) (and subsequent *beginning of contact phase*) (see, Fig. 1B). The splitting of the candy, i.e., *end of contact phase* and *beginning of jaw closing phase* (T3), was determined by a characteristic rapid ramp increase in the vertical jaw movement (jaw closing) which coincided by both a clear sound (≥30% of the loudest signal) and increased EMG activity of the masseter muscles.

### Statistical analysis

The split performance data was analyzed with a general estimating equation for repeated measures. The outcome variable (i.e., mean deviation from ideal split, unsuccessful, failed and perfect splits) was assumed to be of count-variable type and the event rate per 30 trials was analyzed. The link function and the outcome distribution was set to logarithmic and negative binomial respectively. In all these analyses the offset was set to 30. The chosen covariance structure was unstructured. Each statistical model included the prognostic variables gender, age, condition, group, and interaction between condition and group.

Continued data from jaw movements (i.e., peak vertical velocity, position at different time-points; T1-T3, and duration; total duration, jaw opening, contact-establishing and contact phases) were analyzed employing a mixed-effects model for repeated measures^19^. The study design of two within subject effects, repeated measures and conditions makes it suitable to use a combined covariance structure in the analyses. The covariance structure can be described as a combination of unstructured and compound symmetry. The denominator degrees of freedom were computed with the Satterthwaite method and in each analysis the residual assumptions were checked. For each subject, data from all 30 trials were combined to obtain subject mean values and presented as group mean and standard deviation. However, if the data was skewed to the right a logarithmic transformation was done and therefore the results are presented with median and 25–75 percentile. Each statistical model included the prognostic variables gender, age, group, condition and interaction between condition and group.

Split performance (mean deviation from ideal split, unsuccessful, failed and perfect splits) and jaw movements (peak vertical velocity, position at different time-points; T1-T3, and phase durations; total duration, jaw opening phase, contact-establishing phase and contact phase) were further investigated by calculating the relative changes. The relative change expressed in percentage within the group was calculated as the mean difference between the baseline and the intervention divided by mean deviation at baseline and was analyzed with an ordinary least squares analysis taking into consideration heterogeneity of variance across groups. All statistical analyses were carried out in the SAS 9.4 software (SAS Institutet INC., Cary, NC, USA) and the level of significance set at ≤0.05.

## Results

### General observations

During performance of a motor task it is evitable that motor skill can be assessed at the levels of task success and movement quality, but the link between these levels remains poorly understood^20^. In the present study we investigated the perturbation caused by the transient deprivation of sensory input to the PMRs on oral fine motor control and associated jaw movements during a standardized intraoral manipulation of food. When given the instruction to split a chocolate candy into two equal halves, all participants performed the task without any hesitations by moving the lower jaw downward (jaw opening phase) to accommodate the candy. The participants positioned the candy between their front teeth in order to obtain a stable clasp (contact-establishing phase) with assistance from the tongue and lips and subsequently making minor adjustments (contact phase) prior to splitting the candy. All participants completed the manipulation and split task, but, there were certain differences between the experimental and control groups. One participant chose not to participate in the intervention section due to some spontaneous commitment.

**Figure 2:**
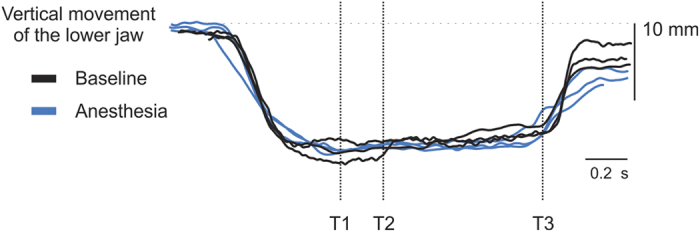
Representative recordings of vertical jaw movement when performing the “manipulation and split” task by three participants in the experimental group at baseline (intact sensory information) and the same participant’s during intervention (no sensory information due to anaesthesia). Notice the similar duration and jaw movement behaviour for the six vertical traces.

During the baseline trials no slippage of the candy was observed in either the experimental or control group. Slippage was characterized by 100% deviation from the ideal split resulting from the escape of the candy from the oral cavity during the attempt of biting it into two equal halves. However, following anesthesia nine of those in the experimental group exhibited at least one or more slippage versus none in the control group. Although, there were some differences in the performance; the vertical jaw movements (i.e., the duration and jaw positions) were almost exactly the same for both the groups at baseline and during intervention. Plotting the raw traces of vertical positions of the jaw during movement for three trials from three different participants during baseline and during intervention in the experimental group shows similar overall pattern regarding the time taken to do the task and the position of the jaw during the task (see, Fig. 2). This observation was not only specific for the represented trials in the figure but was consistent throughout all participants in both the groups between baseline and intervention. All values for both groups (Experimental; *Bas* and *Ane*, and Control; *Bas* and *nAne*; mean (SD) or median (25–75 percentile)) are summarized in Table 1.

### Split performance

#### Ideal split

The largest piece resulting from the split of the candy during the behavioral task was weighted and compared with half the weight of the candy (0.42 g) (Svensson *et al*.^14^, Kumar *et al*.^15^).

**Figure 3:**
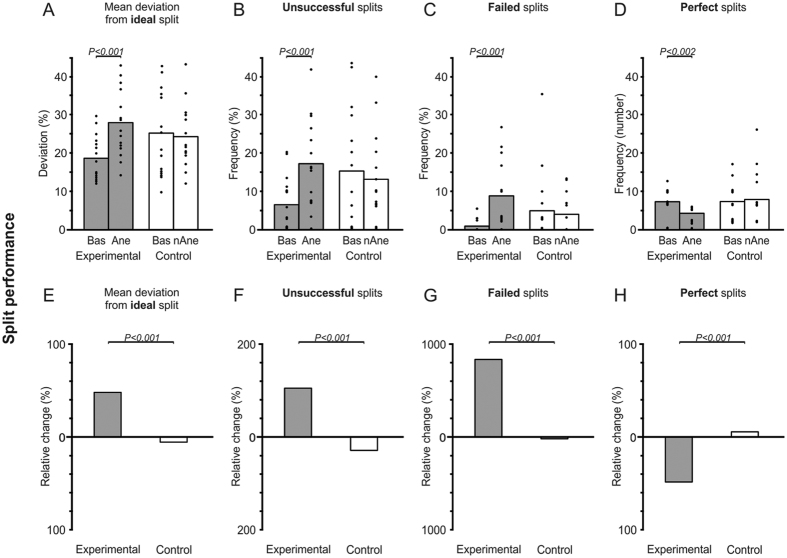
(**A–D**) The split performance reflected by deviations in weight from an ‘ideal’ split for the participants in the *Experimental* group (at baseline (*Bas*) and during intervention (*Ane*)) and *Control* group (at baseline (*Bas*) and during intervention (n*Ane*)). (**A**) Mean percentage weight deviation from an ‘ideal’ split. (**B**) ‘Unsuccessful’ splits: Mean frequency (%) of the splits with a deviation of >50% from the ‘ideal’ split. (**C**) ‘Failed’ splits: Mean frequency (%) of the splits with a deviation of >75% from ‘ideal’ split. ‘Perfect’ splits: Mean frequency (number of occurrences) of splits with a deviation of <5% from ‘ideal’ split. The height of each bar indicates the mean value for all of the participants in a group and the filled circles represent means of thirty splits for individual participants. (**E–H**) The mean relative changes (shown in percentage) for the experimental and control groups for mean deviation from ideal splits, unsuccessful, failed and perfect splits.

The results showed significant effect of condition (P = 0.003) with a significant interaction between group and condition (P < 0.001). Further, performance was impaired in the experimental group with significant increase in deviation (decreased performance) during intervention (*Ane*) compared to baseline (P < 0.001). However, there was no difference in performance between the baseline and no-anesthesia (nAne) in the control group (P = 0.567). Further, the relative changes between baseline (Bas) and anesthesia (Ane) in the experimental group was observed to be significant higher than between baseline (Bas) and no-anesthesia (nAne) in the control group (P < 0.001) (see, Fig. 3A,E).

#### Unsuccessful, failed and perfect splits

Similar to ideal splits the unsuccessful and failed splits showed significant effect of condition (P = 0.009; P = 0.006, respectively) with a significant interaction between group and condition (P = 0.002; P = 0.007, respectively). The frequency of both the unsuccessful and failed splits in the experimental group increased significantly (decreased performance) after intervention compared to baseline (P < 0.001; P < 0.001, respectively) yet, there was no significant difference in the control group (P = 0.138; P = 0.244, respectively) (Fig. 3B,C). Further, there were significant differences in failed splits between the groups at baseline (P = 0.019). There was a significant interaction between group and condition for perfect splits (P = 0.003). The occurrence (frequency) of perfect splits in the experimental group was less during the intervention than baseline (P < 0.002). However, there was no significant difference in the occurrence of perfect splits during intervention than baseline in the control group (P = 0.752) (Fig. 3D). Further, there was a significant increase in the occurrence of unsuccessful (P < 0.001) and failed splits (P < 0.001) and a significant decrease in the occurrence of perfect splits (P < 0.001) (Fig. 3F–H) when the relative changes in the experimental and control groups were compared.

### Jaw movements

#### Positions at different time points

**Figure 4:**
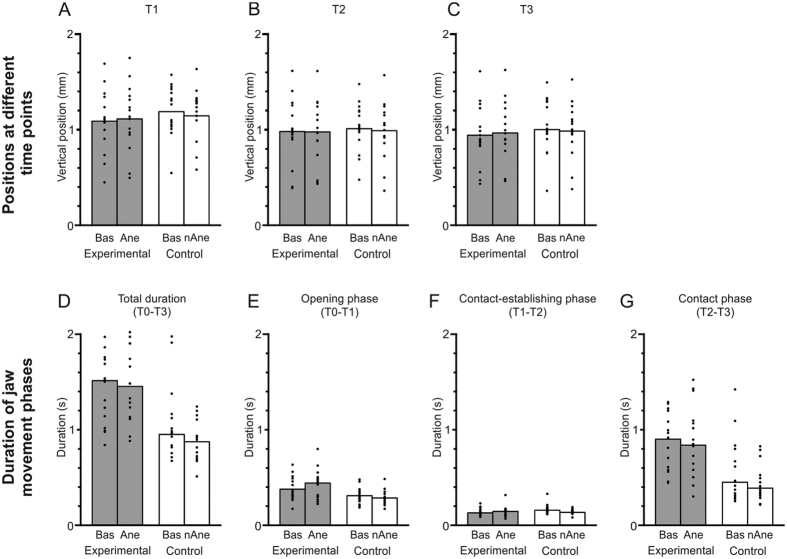
(**A–C**) The jaw movements (position at different time-points) for the participants (30 in total, 15 in each group) in the *Experimental* group (at baseline (*Bas*) and during intervention (*Ane*)) and *Control* group (at baseline (*Bas*) and during intervention (n*Ane*)). (**A–C**) Mean vertical positions of the jaw at time-points; T1, T2 and T3. The height of each bar indicates the mean value for all of the participants in a group and the filled circles represent means of thirty splits for individual participants. (**D–G**) Total task and phase durations for the participants (30 in total, 15 in each group) in the *Experimental* group (at baseline (*Bas*) and during intervention (*Ane*)) and *Control* group (at baseline (*Bas*) and during intervention (n*Ane*)). (**D**) Total duration of the task (T0-T3). (**E**) Duration of the opening phase (T0-T1). (**F**) Duration of the contact-establishing phase (T1-T2). (**G**) Duration of the contact phase (T2-T3). The height of each bar indicates the median value for all of the participants in a group and the filled circles individual mean of all splits.

The vertical position of the jaw during movement showed significant effect of groups with no significant effect of condition and no interaction between groups and condition at T1 (P = 0.001; P = 0.860; P = 0.655, respectively), T2 (P = 0.005; P = 0.810; P = 0.829, respectively) and T3 (P = 0.013; P = 0.547; P = 0.544, respectively) (Fig. 4A–C). However, there was no significant difference in the relative changes between the groups at T1 (P = 0.748), T2 (P = 0.859) and T3 (P = 0.112).

#### Velocity and duration of jaw movement phases

The peak vertical velocity (during jaw opening phase) and the total duration of the jaw during movements showed significant effect of groups (P < 0.001, P < 0.001, respectively) but no significant effect of condition (P = 0.534; P = 0.360, respectively) with no significant interaction (P = 0.788; P = 0.196, respectively) (see, Fig. 4D for total duration). Further, there were also no significant differences in the relative changes of the peak vertical velocity, between the groups (P = 0.249; P = 0.498, respectively).

The jaw opening phase (T0-T1) and contact phase (T2-T3) showed a significant effect of groups (P = 0.004, P < 0.001; respectively) but neither a significant effect of condition (P = 0.557, P = 0.360; respectively) nor a significant interaction (P = 0.311, P = 0.196; respectively). However, the contact establishing phase (T1-T2) showed neither a significant effect of groups (P = 0.291) nor significant effect of condition (P = 0.296) or a significant interaction (P = 0.184) (see, Fig. 4E–G). Further, there was also no significant difference in the relative changes between baseline and intervention in the experimental and control groups; for jaw opening phase (P = 0.192), contact-establishing phase (P = 0.132) and contact phase (P = 0.384).

## Discussion

The “manipulation and split” task has proven to be a useful tool for studying oral fine motor control^14^,^15^,^21^. In the present study it was decided to study the effect of somatosensory perturbation caused by the deprivation of sensory information from the PMRs on oral motor control during intraoral manipulation of food morsels. The results of the present study revealed that there were significant changes in performance of this oral fine motor task due to anesthesia, however, no effect were observed on the jaw movements with respect to the vertical jaw position and duration. Previously, we have observed that the sensory information provided by the PMRs plays an important role in positioning the spherical candy between the front teeth and subsequently splitting it with greater accuracy. Lack of such information, e.g., in individuals with fixed implant-supported prostheses results in poorer performance and altered motor behavior during the task^14^. The reasons for such differences in behavior and oral motor control will be discussed in the following paragraphs.

### Performance during intraoral manipulation of food

It has previously been shown that PMRs play a pivotal role in controlling and directing the forces needed to hold the food morsel and the control of these forces was shown to be disrupted during periodontal anesthesia^9^,^22^. In the present experiment, anesthetizing the incisors perturbed the oral sensory motor control process reflected in a decreased performance and an increase in number of unsuccessful and failed splits, with a subsequent decrease in the occurrence of perfect splits during the periodontal anesthesia. The results confirm previous findings that the PMRs play an important role in oral motor control and that loss or reduced inputs from the PMRs (after local anesthesia injection to the upper and lower incisors) cannot be fully compensated by inputs from other orofacial mechanoreceptors (e.g., mechanoreceptors in oral mucosa, muscle spindles or temporomandibular joint, etc.). Apparently, such a lack of peripheral afferent input to the motor cortex attenuates fine-motor control of the jaws, as also previously demonstrated in connection with a hold-and-split task^9^,^22^,^23^. The hold-and-split task and its relationship to the manipulation and split task have not been addressed.

The task performance in the present study, under the influence of anesthesia was comparable to the task performance by patients with fixed implant-supported prostheses^14^. It was previously suggested that the participants with the loss of PMRs (for example; patients with fixed implant-supported prostheses) behave like participants with acute periodontal anesthesia in some aspects of biting behavior^9^,^22^. Participants with anesthesia, similar to patients with fixed implant-supported prostheses, may thus rely (even though not fully compensate) on adjacent or other types of orofacial mechanoreceptors for sensory inputs which explains the relatively poor performance in fine motor tasks in this group^14^. In agreement with this prediction, 28% deviation from the ideal split was observed due to anesthesia in the experimental group which was similar to the performance exhibited by the individuals with fixed implant-supported prostheses (28%)^14^. Similarly, the occurrence of unsuccessful and failed splits due to loss of sensory information from the PMRs (in the experimental group during intervention) increased (17% and 8%, respectively), and it was almost comparable to the previous study (fixed implant-supported prostheses; 24% and 8%, respectively). In the present study, the number of perfect splits decreased due to anesthesia for the participants in the experimental group. It may also be noticed that there was no significant difference in the baseline and no-anesthesia in the control group in any of the above mentioned parameters.

The results have also shown significant differences in the occurrence of failed splits at baseline between the two groups and significant main effects of group for jaw movement positions (i.e., T0, T1 and T2) and jaw movement durations (total duration, jaw opening and contact phase). However, there was a drastic decrease in performance as demonstrated by a 47% increase in the deviation from the ideal split in the experimental group. Subsequently, there was no major change in performance as indicated by a meagre 4% increase in the deviation in the control group (see, Fig. 3E). It is suggested that the baseline differences in performance between the experimental and control group could be attributed to inter-individual variations in motor performance. We have previously observed baseline differences in different groups across different studies, for example Kumar *et al*.^15^ and Zhang *et al*.^21^. Possibly, there is a large variation in motor performance in the general population and these intergroup differences can be attributed to the above mentioned factors. However one limitation of the study was that the allocation of the participants in the two groups was not randomized and this lead to unequal distribution of males and females in the two groups. Therefore, these factors may have resulted in the large group differences observed in the baseline values. Further research should explore the potential reasons for individual differences in fine motor tasks but the practical suggestion would be to evaluate relative changes to circumvent potential baseline differences in performance.

### Jaw movements

Decreases in the velocity of jaw movements have been suggested to indicate fatigue, pain or altered function^24^. In the current study no change in the peak vertical velocity was observed (during jaw opening phase) in the experimental and control group which may indicate no effect of anesthesia on the general stomatognathic function. The vertical position of the jaw at T1, T2 and T3 were also virtually identical for the two groups and conditions. Similar observations are also evident in individuals with fixed implant-supported prostheses in both the upper and lower jaw^14^. This shows that the relative precision of the jaw positioning is not hampered due to absence of sensory information from PMRs, due to anesthesia.

The “manipulation and split” task used in the current study similar to most manual task involves a sequence of action phases that are required to achieve the task sub-goals and also for overall successful completion of the task. As suggested above, the successful completion of these sub-goals will be typically dependent on discrete sensory signals from peripheral receptors^25^,^26^. At the same time, muscle commands required to execute an action can also be launched in anticipation of the upcoming movement^27^,^28^. The brain predicts an outcome of the movement and identifies the optimal motor commands to achieve the objective of the task. Such predictions can be acquired and updated by previous experience (learning) and may also aid in optimizing motor performance^27^,^29^. In the present study the duration of the overall task and of the individual phases of jaw movement did not differ between groups and conditions. There was also no significant change in the jaw opening and the contact-establishing phase after anesthetic injection which is in agreement with the behavior of implant-supported prosthetic patients who did not differ from the natural dentate participants in terms of these parameters^14^.

In addition, during the contact phase, when the candy is placed between the teeth, the participants with fixed implant-supported prostheses surprisingly take shorter time (in contrary to individuals with natural dentition) to perform the task^14^. Therefore, participants with fixed implant-supported prostheses exhibit an altered motor behavior where they do not rely on the sensory information in the absence of PMRs and hence, complete the manipulation task with relatively shorter duration (due to shorter contact phase) but poorer performance. Consequently, it might be anticipated that deprivation (due to anesthesia) of sensory inputs in natural dentate subjects would result in significant changes in the total duration of the task specifically the contact phase. However, in the present study there were no significant effects of anesthesia on the contact phase indicating that transient deprivation of sensory inputs does not alter the action phases related to the task; contrary to our hypothesis. Further, since the participants in the present study have participated in the earlier published study where the participants demonstrated increase in precision of task performance, and optimization of jaw movements in terms of reduction in duration of various phases of jaw movements, due to repeated performance of the task for about thirty minutes^15^. Hence, it can be hypothesized that these subjects may have auto-regulated the motor commands where sequential events of task sub-goals could be triggered due to optimized performance, despite the decreased sensory input.

Our results on oral motor control did not corroborate the observations in general motor control from previous studies^30^,^31^. These studies on grip forces with digits have shown reduced performance and loss of automatic triggering of movements by other sensory inputs, sequential to the motor task^30^,^31^. Absence of sensory inputs enhances the mental attention required to complete the restrained task thereby increasing the duration of task^31^. However, we have not observed the increase in the duration of task in compromised sensory conditions due to local anesthesia in the current study. It is suggested that the two systems (i.e., trigeminal and spinal system) can have several inherent differences wherein visual feedback is available in the hand motor tasks and absent during oral tasks^32^. Studies have emphasized that role of visual feedback as one of the important modality for optimizing performance^33^,^34^. Subsequently, absence of sensory inputs motor performance is impaired and mental attention required to complete the restrained task is prolonged. It was previously observed that digital anesthesia without visual feedback caused tremendous impairment of digital motor control as compared to performance with feedback^34^,^35^,^36^,^37^. Therefore, the differences between these findings and our present results may be attributed to the inherent differences in the motor systems.

In conclusion, transient deprivation of sensory information from PMRs attenuates the oral motor control, resulting in poorer performance during the manipulation and split task, yet no significant changes was observed in the duration of jaw movements. These observations indicate that the participants are able to open their jaws with equal speed, grasp the candy efficiently but not split it accurately due to the influence of anesthesia. These findings may be important because the topic of orofacial motor skill acquisition following cortical damage or manipulation of sensory inputs due to for example extraction of teeth, prosthetic rehabilitation or a nerve injury of the patients with extracted teeth has received little attention and more research is needed in this direction^38^,^39^. It has been emphasized previously that elucidation of the acquisition of skills following a change in the oral sensory environment would provide key insights into successful rehabilitation after such a change.

## Additional Information

**How to cite this article**: Grigoriadis, J. *et al*. Perturbed oral motor control due to anesthesia during intraoral manipulation of food. *Sci. Rep.* **7**, 46691; doi: 10.1038/srep46691 (2017).

**Publisher's note:** Springer Nature remains neutral with regard to jurisdictional claims in published maps and institutional affiliations.

## Figures and Tables

**Table 1:** Mean values for the parameters performance and jaw movements associated with the “manipulation and split” task under the different conditions.

		Experimental Group			Control Group			*P*-value (relative change between groups)
		Baseline (n = 15)	Anesthesia (n = 15)	Relative change	Baseline (n = 15)	No-anesthesia (n = 15)	Relative change	
**Performance**	Deviation from ideal split (%)	18.9 (6)	27.9 (9)	+48%	25.4 (13)	24.3 (9)	−4%	P < 0.001
	Unsuccessful split (%)	7.3 (3)	17.3 (7)	+137%	15.8 (4)	12.9 (17)	−18%	P < 0.001
	Failed split (%)	0.9 (3)	8.2 (2)	+811%	5.3 (2)	3.6 (10)	−32%	P < 0.001
	Perfect split (n)	7.1 (1)	3.8 (4)	−46%	7.1 (2)	7.6 (5)	+7%	P < 0.001
**Jaw movements**	Peak vertical velocity (mm/s)	52.9 (2)	54.7 (2)	+3%	68.9 (2)	73.6 (2)	+7%	P = 0.249
	T1 position (mm)	10.9 (3)	11.1 (3)	+2%	11.9 (1)	11.4 (1)	−4%	P = 0.748
	T2 position (mm)	9.8 (3)	9.7 (4)	−1%	10.2 (3)	9.9 (3)	−3%	P = 0.859
	T3 position (mm)	9.4 (3)	9.7 (3)	+3%	10.1 (3)	9.8 (3)	−3%	P = 0.112
	Total duration (s)	1.53 (1.1–1.7)	1.42 (1.2–1.7)	−7%	0.95 (0.8–1.1)	0.90 (0.7–1.0)	−5%	P = 0.498
	Jaw opening phase (s)	0.38 (0.3–0–5)	0.45 (0.3–0.5)	+18%	0.29 (0.3–0.4)	0.26 (0.2–0.3)	−10%	P = 0.192
	Contact-establishing phase (s)	0.13 (0.1–0.2)	0.14 (0.1–0.2)	+8%	0.15 (0.1–0.2)	0.14 (0.1–0.2)	−7%	P = 0.132
	Contact phase (s)	0.91 (0.6–1.1)	0.84 (0.6–1.1)	−8%	0.47 (0.3–0.7)	0.39 (0.3–0.6)	−17%	P = 0.384

Mean values of measured parameters of performance and jaw movements for the participants (n = 30 in total, 15 in each group) in the *Experimental* group (at baseline (*Bas*) and during intervention (*Ane*)) and *Control* group (at baseline (*Bas*) and during intervention (n*Ane*)). Data are presented as group mean and standard deviation (mean (SD)), however, data for total duration, jaw opening phase, contact-establishing phase and contact phase in the form of median with 25–75 percentile (median (25–75 percentile)). Relative change and the significance (p-value) are presented between the experimental and control group.
